# Supplementary material for: Isoprene Oxidation by the Gram-Negative Model bacterium Variovorax sp. WS11
Source: Microorganisms. 2020 Feb 29;8(3):349. doi: 10.3390/microorganisms8030349 (PMC7143210; doi:10.3390/microorganisms8030349)
Supplement: Supplementary file 1 [file microorganisms-08-00349-s001.zip › microorganisms-730286-proofreading sup/Title.docx]

Figure S1: Schematic of an isoprene-fed fermentor;

Figure S2: Phylogenetic relationship of Variovorax sp. WS11 to other Variovorax spp.;

Figure S3: Putative oxygenase gene clusters in the genome of Variovorax sp. WS11;

Figure S4: Expression of isoG induced by isoprene and epoxyisoprene;

Figure S5: Expression of isoA when grown on isoprene, glucose, or a combination of glucose and isoprene;

Table S1: Features of the Variovorax sp. WS11 genome;

Table S2: Profile of carbon sources capable of sustaining growth of Variovorax sp. WS11;

Table S3: Comparison of translated iso genes by BLASTp.
